# Oral somatosensory alterations and salivary dysfunction in head and neck cancer patients

**DOI:** 10.1007/s00520-023-08086-7

**Published:** 2023-10-13

**Authors:** Reisya Rizki Riantiningtyas, Alexandre Valenti, Anestis Dougkas, Wender L. P. Bredie, Camille Kwiecien, Amandine Bruyas, Agnès Giboreau, Florence Carrouel

**Affiliations:** 1 Institute Paul Bocuse Research Centre, 69130 Ecully, France; 2 Health Systemic Process (P2S), Research Unit UR4129, University Claude Bernard Lyon 1, University of Lyon, 69008 Lyon, France; 3 Section for Food Design and Consumer Behaviour, Department of Food Science, Faculty of Science, University of Copenhagen, 1958 Frederiksberg C, Denmark; 4 Laboratoire Centre Européen Nutrition Et Santé (CENS), CarMeN, Unité INSERM 1060, Université Claude Bernard Lyon 1, 69310 Pierre-Bénite, France; 5 Danone Nutricia Research, 3584 CT Utrecht, The Netherlands; 6 Institute of Cancerology, Hospices Civils de Lyon, Hôpital Croix Rousse, 69004 Lyon, France

**Keywords:** Oral somatosensation, Salivary function, Head and neck cancer, Oral tactile sensitivity, Food texture sensitivity, Thermal sensitivity, Chemesthetic sensitivity

## Abstract

**Purpose:**

Patients with head and neck cancer (HNC) are at high risk of malnutrition due to eating difficulties partly mediated by sensory alterations and salivary dysfunction. Clinical studies have mostly focused on taste and smell alterations, while changes in oral somatosensory perception are largely understudied. The study aimed to investigate oral somatosensory (tactile, texture, chemesthetic, and thermal) responses and salivary functions of HNC patients in comparison to healthy controls.

**Methods:**

A cross-sectional study was conducted using psychophysical tests in HNC patients (*n* = 30) and in age- and gender-matched control subjects (*n* = 30). The tests included measurements of point-pressure tactile sensitivity, whole-mouth chemesthetic stimulation, food texture discrimination, and temperature discrimination. Salivary functions, including hydration, saliva consistency, pH, volume, and buffering capacity, were also evaluated.

**Results:**

HNC patients demonstrated significantly lower chemesthetic sensitivity (for medium and high concentrations, *p* < 0.05), thermal sensitivity (*p* = 0.038), and salivary functions (*p* = 0.001). There were indications of lower tactile sensitivity in the patient group (*p* = 0.101). Patients were also less sensitive to differences in food roughness (*p* = 0.003) and firmness (*p* = 0.025).

**Conclusion:**

This study provided evidence that sensory alterations in HNC patients extend beyond their taste and smell. The measurements demonstrated lower somatosensory responses, in part associated with their reduced salivary function. Oral somatosensory alterations and salivary dysfunction may consequently impart the eating experience of HNC patients. Thus, further investigations on food adjustments for this patient group seem warranted.

**Supplementary Information:**

The online version contains supplementary material available at 10.1007/s00520-023-08086-7.

## Introduction

An estimated 747,000 new cases of head and neck cancer (HNC) occurred worldwide in 2020 [1]. Due to the cancer site, HNC patients are at higher risk of malnutrition, with the prevalence of malnutrition among this population estimated to be 74% [2]. HNC patients experience physiological changes that contribute to eating difficulties such as food-related sensory alterations and salivary dysfunction [3–5]. These side effects were experienced by 70–90% of HNC patients undergoing radiotherapy and continued to persist in some of the patients 1–2 years post-treatment [3, 6, 7]. These altogether influenced their eating experience, resulting in weight loss and a negative impact on their quality of life [8–10].

Altered sensory perceptions are associated with diminished eating pleasure, loss of appetite, and changes in food choices [8, 11, 12]. Sensory perception is a multimodal process involving the gustatory/taste, olfactory/smell, and somatosensory systems [13]. The somatosensory system comprises multiple sub modalities detecting and translating mechanical, thermal, and nociceptive stimulations throughout the oral epithelium into the perception of texture, temperature, and chemesthesis (e.g. spiciness of chili, cooling of mint) [14, 15]. In addition, saliva serves several functions that influence patients’ eating experience including food flavour release and perception, facilitation of chewing and swallowing, lubrication, and cleansing of the oral cavity [16, 17].

Studies among HNC patients have focused on examining chemosensory alterations (i.e., taste and smell). The prevalence of taste alterations among radiated HNC patients was estimated to be 79%, with the prevalence of long-term alterations at 23–53% while smell alterations were reported by 30–60% of HNC patients [18, 19]. These reported changes in taste and smell are clear indicators of orosensory changes and may also relate to changes in somatosensory perception and mouthfeel as they share similar oral tissues. A few studies have reported on one or two sub modalities of the somatosensory mechanisms [20–22]. Others reported on altered perceptions of food texture, temperature, and chemesthetic sensations and their influence on the eating behaviour of HNC patients using subjective measurements [23, 24]. The present study aimed to reveal the extent to which changes in oral somatosensory perception and salivary functions occur in HNC patients using a set of objective sensory measurements. These findings will provide further insights into the underlying mechanisms of altered food perception in this patient group.

## Materials and methods

### Study design and setting

The study was a part of a cross-sectional study (Somestalim) conducted in accordance with the Declaration of Helsinki, approved by the Personal Protection Committee of Ile-de-France (RCB N° 2021-A02961-40), and registered to the Clinical Trials Registry (NCT05272917). The patient group consisted of HNC patients recruited during their outpatient consultations at the Hospices Civils de Lyon (France) by clinical research associates or physicians. The control group consisted of healthy volunteers matched in terms of sex and age, recruited from Ecully (France) through advertisements via flyers and newsletter e-mails. Informed consent was obtained from all participants. The present paper was written in accordance with the STROBE guidelines (Supplementary Table S1).

### Study participants

Patients were eligible if they fulfilled the following criteria: age between 18 and 70 years old, diagnosed with tumours of the upper aerodigestive tract (including oral cavity, pharynx, and larynx), salivary glands, maxillary sinuses, or nasopharynx, treated by radiotherapy alone in combination with systemic treatment, surgery, or both. The radiotherapy must have been completed between 4 months to 5 years ago. Controls were healthy volunteers matched in sex and age (± 5y). For all participants, the exclusion criteria were as follows: pregnant or breastfeeding, known food allergy or intolerance, inability to swallow soft food, restricted mouth opening (trismus), and a lack of tongue mobility (unable to extend the tongue or large tongue resection).

### Outcomes

The outcomes were comparisons of somatosensory responses (tactile, texture, chemesthetic, and thermal sensitivity) and salivary function between HNC patients and controls.

### Study procedure

**Fig. 1:**
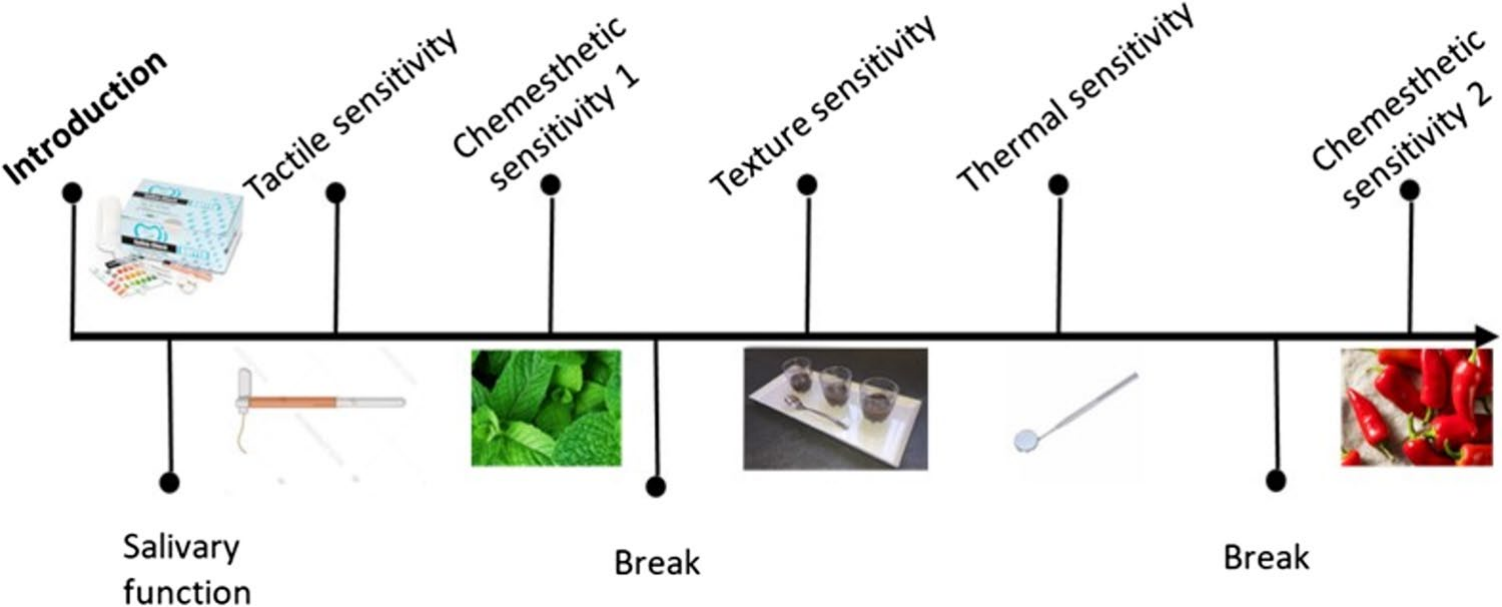
Overview of the study visit, including the order of tests

The study consisted of a single visit (± 1.5 h) which took place at Croix Rousse and Lyon-Sud hospitals (Lyon, France) for the patient group and at the Institute Paul Bocuse research centre (Ecully, France) for the control group, between May 2022 and April 2023. Participants were informed to refrain from eating, drinking, and smoking 1 h before the visit. The visit commenced with a verification of the eligibility criteria followed by a detailed explanation of the procedure (Fig. 1). Then, participants were asked to complete their sociodemographic information and medical history. Participants performed the salivary function test, followed by the different psychophysical tests**.**

#### Analysis of salivary function

The salivary function test was performed using Saliva-Check BUFFER kit (GC Europe, Sucy-en-Brie, France). The test aimed to measure hydration, saliva consistency, pH, volume, and buffering capacity. All tests were performed according to the instructions of the manufacturer. First, the unstimulated saliva was analysed. Hydration was assessed, after drying the labial mucosa with gauze and subsequent measuring of the time taken for new saliva droplets to appear (< 60 s: normal, > 60 s: low). The consistency was classified as clear/watery, frothy/bubbly, or sticky/frothy following visual observation of saliva at the back of the mouth. The pH of unstimulated saliva was determined using pH paper (pH 6.8–7.8: normal, 6.0–6.8: moderately acidic, 5.0–5.8: very acidic). Stimulated saliva was then analysed. Stimulated saliva flow corresponds to the volume of saliva collected for the 5 min during which patients chewed a paraffin tablet (> 5 ml: normal, 3.5–5 ml: low, < 3.5 ml: very low). The buffering capacity was determined by depositing stimulated saliva on a test strip provided in the kit.

#### Analysis of oral tactile sensitivity

The tactile sensitivity on the tongue was determined with a point-pressure test using Von Frey monofilaments (Aesthesio®, San Jose, USA). The test was performed with three different sizes of monofilaments representing forces of 0.008, 0.02, and 0.04 g [25]. Participants were blindfolded and asked to respond to whether they could detect a touch on the tongue apex. A balanced number of true and false touch exposures (5 each) were randomly presented for each monofilament. In addition to identifying the tactile stimulus (present/absent), participants were asked to indicate the degree of certainty of their response (sure/unsure). R-index was calculated as an estimated probability of correctly identifying the target touch stimulus from the presentation of the blank stimulus (no touch), representing an index of their tactile sensitivity [25].

#### Analysis of chemesthetic sensitivity

##### Preparation of menthol and capsaicin solutions

Menthol and capsaicin were selected to evaluate sensitivity to cooling and pungent sensations, respectively. The menthol and capsaicin solutions were made from single stock solutions. L-menthol (≥ 99%, Sigma-Aldrich, Steimheim, Germany) and natural capsaicin (#360376, Sigma-Aldrich, Steimheim, Germany) were first dissolved in 96% ethanol (EMSURE®, Sigma-Aldrich, Steimheim, Germany). These stocks were diluted with water to reach the final concentrations (Table 1) and supplemented with ethanol to standardise all stimuli to equal ethanol concentration of 0.5% (v/v) for menthol and 0.1% (v/v) for capsaicin, as ethanol may also elicit chemesthetic stimulation. The preparation procedure including the concentrations referred to a previous study [26] followed by a series of pilot tests.

**Table 1:** Sample series for chemesthetic sensitivity test with menthol and capsaicin solutions

Chemesthetic modality	Chemical compound	Concentrations (ppm)		
		Low	Medium	High
Cooling	Menthol	7.8	31.3	125.0
Pungency/spiciness	Capsaicin	0.1	1.0	10.0

##### Whole-mouth stimulation test

Whole-mouth stimulation tests using menthol and capsaicin solutions at varying concentrations were used to assess chemesthetic sensitivity. Using the sip-and-spit procedure, participants were asked to sip the entire solution (10 mL) and expectorate it after 10 s. After another delay of 10 s, participants rated the perceived intensity on a 100-mm general labelled magnitude scale (gLMS). The solutions were presented in increasing order of concentration and a break of 3–4 min was held between evaluations to avoid adaptation to the stimulus. The cooling and pungent sensations were evaluated at different sessions of the experimental procedure (Fig. 1) to avoid cross-adaptation.

#### Analysis of food texture sensitivity

##### Preparation of food samples

Three sets of chocolate mousse with three different levels of firmness, thickness, or roughness were prepared to assess food texture sensitivity. First, a chocolate milk base was prepared with 800 g of whole-fat milk (UHT), 200 g of chocolate (Carraibe 66% cacao, Valrhona), and 100 g of granulated sugar. These ingredients were mixed on medium heat until fully homogenised.

Firmness samples were produced by dissolving the respective amount of agar (Texturas gelification agar, Albert y Ferran Adria) as indicated in Table 2 into the chocolate milk base, then mixing on medium heat until boiling. The mixtures were poured into containers and cooled into a gel consistency. The same procedures were followed to produce thickness samples but once gelified, samples were blended into puree using a food processor. The roughness samples were similarly produced by mixing the chocolate milk base with 0.5% (w/w) of agar and blended upon gelification. Then the respective amount of wheat fibre (Jelucel® WF 90, provided by Jeluwerk, Rosenberg, Germany) as indicated in Table 2 was incorporated into the mixture. Wheat fibre is insoluble in water, therefore elicited a sensation of roughness when incorporated into the mousse.

**Table 2:** Sample series for food texture sensitivity test with chocolate mousse samples varying in firmness, thickness, or roughness

Texture attribute	Mechanical treatment	Added ingredient	Concentrations of added ingredients (w/w)		
			Low	Medium	High
Firmness	Different concentrations of agar are added and allowed to gelify	Agar	0.50%	0.75%	1.0%
Thickness	Same procedure as firmness samples, but samples are blended upon gelification	Agar	0.50%	0.75%	1.0%
Roughness	Same procedure as low thickness sample, but wheat fibres were added	Wheat fibre	0%	2.0%	4.0%

##### Texture discrimination test

A texture discrimination test using the chocolate mousse samples was used to determine food texture sensitivity. Participants were first asked to taste the samples and rank them in increasing order, based on the texture attributes of the set (firm/thick/rough). The accuracy in ranking the samples was used to calculate the percentage of correct responses, in each attribute. Next, participants were asked to rate the intensity of the texture attributes on a 100-mm visual analogue scale anchored by the terms “not at all” and “extremely”. The presentation order of the sets and samples was randomised for each participant.

#### Analysis of thermal sensitivity

A temperature discrimination test using metal dental mirrors immersed in water maintained at temperature of 3, 20, or 55 °C was used to assess thermal sensitivity [27]. The back of the dental mirror was placed in contact with the centre of the tongue for 1 s. Blindfolded participants had to indicate the thermal sensation that was perceived (cold/neutral/hot), from which the percentage of correct responses was calculated. Each temperature was presented 3 times in a randomised order.

### Statistical analyses

Sample size calculation was based on a previous study using tactile sensitivity as the outcome measure with an α risk of 0.05, power 1-β of 80%, effect size of 0.8, standard deviation of 0.7, and delta of 0.37 [28] which lead to a minimum of 29 participants per group. SPSS Statistics 23 (IBM Corporation) was used to perform statistical analyses. Descriptive statistics are presented as mean ± SD or percentage. Comparisons between the patient and control group were analysed using an independent *t*-test (continuous) or chi-square test (categorical). Significant level was set at *p* = 0.05.

## Results

### Characteristics of the study population

In total, 30 patients and 30 controls participated in the study. Sex and age (± 5 y) were individually matched between the patient and control. All patients received radiotherapy, 70% of the patients had surgery, and 47% had systemic treatment. Table 3 shows the characteristics of the participants in the patient and control groups.

**Table 3:** Demographic and clinical characteristics of patients and sex and age-matched healthy controls, n (% ^*a*^)

Variable	Patient group (*n* = 30)	Control (*n* = 30)
Age (mean ± SD)	59.9 ± 7.5	59.7 ± 6.8
Sex		
* Male*	23 (77)	23 (77)
* Female*	7 (23)	7 (23)
Household		
* Alone*	6 (20)	7 (23)
* Living with partner/ children*	23 (7)	23 (7)
* Other*	1 (3)	0 (0)
Smoking status		
* Current smoker*	6 (20)	2 (7)
* Former smoker*	4 (13)	6 (20)
* Non-smoker*	20 (67)	22 (73)
Clinical characteristics		
Primary tumour site		
* Oropharynx*	17 (57)	-
* Hypopharynx*	2 (7)	
* Nasopharynx*	2 (7)	-
* Oral cavity*	6 (20)	-
* Larynx*	3 (10)	-
Histologic type		
* Squamous cell carcinoma*	26 (87)	-
* Other*	4 (13)	-
Tumour stage		
* I*	0 (0)	-
* II*	3 (10)	-
* III*	13 (43)	-
* Iva*	9 (30)	-
* IVb*	2 (7)	-
* N/a*	3 (10)	-
Types of treatment		
* Radiation*	2 (7)	-
* Radiation* + *surgery*	14 (47)	-
* Radiation* + *surgery* + *systemic treatment*	7 (23)	-
* Radiation* + *systemic treatment*	7 (23)	-
Duration since the end of radiotherapy		
< *1 year*	11 (37)	-
> *1 year*	19 (63)	-

^*a*^ The sum of percentages may be dissimilar to 100% due to rounding

### Measurements of oral somatosensory responses

Somatosensory responses of the two groups are presented in Table 4. The tactile sensitivity in the patient group did not differ significantly compared to the control across all filament sizes 0.04 g (*p* = 0.171), 0.02 g (*p* = 0.329), and 0.008 g, (*p* = 0.101). The texture sensitivity for the chocolate mousses differed between the two groups. The patient group was significantly less sensitive to the differences in roughness compared to the control (*p* = 0.003). Patients rated the samples to be higher in roughness compared to controls, with 17% of patients perceiving the samples to be identical to each other. The patient group was also significantly less sensitive to the differences in firmness compared to the control group (*p* = 0.025). Patients showed a tendency to perceive the samples to be less firm compared to controls, with 10% of patients reported perceiving the samples to be identical to each other. In terms of discrimination ability to thickness, no significant difference was observed between the two groups (*p* = 0.587).

**Table 4:** Somatosensory responses of HNC patients in comparison to controls

Somatosensory responses	Patient group	Control	*p*-value
Oral tactile sensitivity (R-index)			
* 0.008 g filament*	0.73 ± 0.22	0.79 ± 0.15	0.171
* 0.02 g filament*	0.81 ± 0.16	0.85 ± 0.20	0.329
* 0.04 g filament*	0.85 ± 0.15	0.92 ± 0.14	0.101
Food texture sensitivity			
Roughness			
Discrimination task (% correct response)	66.7 ± 42.9	93.3 ± 20.3	0.003
Intensity scaling task (mm)			
* Low roughness*	17.9 ± 16.5	8.0 ± 6.9	0.002
* Medium roughness*	36.9 ± 22.6	27.9 ± 16.2	0.089
* High roughness*	55.3 ± 25.3	54.3 ± 21.8	0.853
Firmness			
Discrimination task (% correct response)	76.7 ± 34.1	93.3 ± 20.3	0.025
Intensity scaling task (mm)			
*Low firmness*	27.8 ± 18.8	31.66 ± 21.4	0.266
* Medium firmness*	57.5 ± 20.2	63.21 ± 20.1	0.193
* High firmness*	68.0 ± 22.3	79.24 ± 11.9	0.018
Thickness			
Discrimination task (% correct response)	90.0 ± 26.5	93.3 ± 20.3	0.587
Intensity scaling task (mm)			
* Low thickness*	18.9 ± 14.6	15.6 ± 10.2	0.303
* Medium thickness*	41.5 ± 16.8	50.5 ± 16.8	0.031
* High thickness*	67.4 ± 16.0	66.6 ± 16.9	0.969
Chemesthetic sensitivity			
Cooling sensation (gLMS)			
*Menthol low*	5.0 ± 5.4	7.10 ± 6.0	0.169
* Menthol medium*	13.3 ± 9.7	19.57 ± 8.9	0.011
* Menthol high*	26.8 ± 13.5	34.37 ± 13.6	0.034
Spiciness sensation (gLMS)			
* Capsaicin low*	3.1 ± 4.4	2.8 ± 3.4	0.745
* Capsaicin medium*	17.2 ± 12.9	28.6 ± 13.7	0.002
* Capsaicin high*	54.2 ± 23.2	65.7 ± 19.2	0.044
Thermal sensitivity (% correct response)	94.1 ± 10.4	98.5 ± 4.8	0.038

Values are expressed as means ± SD, *p* < 0.05: significant difference on independent *t*-test

Patients perceived the chemesthetic solutions to be less intense compared to the control group (Table 4). Significant differences were observed in the medium and high concentrations for both menthol (*p* = 0.011 and *p* = 0.034) and capsaicin (*p* = 0.002 and *p* = 0.044) solutions. For both chemesthetics, the sensory threshold did not seem to be affected; however, in the range above sensory detection, the dose-responses relationship showed a significant decline for the patient group. The thermal sensitivity measured as physical-induced sensation (cold/warm) demonstrated a lower accuracy for the patient group in discriminating these sensations (*p* = 0.038), although they still showed a general good ability to discriminate cold/warm stimuli.

### Measurements of salivary functions

Measurements of salivary functions between the two groups are presented in Table 5. Patients demonstrated significantly lower salivary function compared to the controls (*p* = 0.001). Patients had lower scores for hydration (*p* = 0.002) and stimulated salivary volume (*p* = 0.001), while displaying higher values for saliva consistency (*p* = 0.004). Most participants had an acidic salivary pH of 5.0–6.6 and a normal buffering capacity of 10.0–12.0, with no significant differences between the patient and control groups.

**Table 5:** Salivary functions of HNC patients in comparison to controls, n (% ^*a*^)

Somatosensory responses	Patient group (*n* = 30)	Control (*n* = 30)	*p*-value
Salivary function score (mean ± SD)	10.6 ± 2.6	12.7 ± 2.0	0.001
Hydration			
* Low*	12 (40)	2 (7)	0.002
* Normal*	18 (60)	28 (93)	
Consistency			
* Sticky and frothy*	16 (53)	5 (17)	0.004
* Frothy and bubbly*	8 (27)	8 (27)	
* Clear and watery*	6 (20)	17 (57)	
Saliva pH			
* Very acidic*	5 (17)	2 (7)	0.329
* Moderately acidic*	15 (50)	20 (67)	
* Normal*	10 (53)	8 (27)	
Stimulated saliva volume			
* Very low*	12 (40)	3 (10)	0.001
* Low*	9 (30)	4 (13)	
* Normal*	9 (30)	23 (77)	
Buffering capacity			
* Very low*	3 (10)	2 (7)	0.610
* Low*	5 (17)	8 (27)	
* Normal*	22 (73)	20 (67)	

^*a*^ The sum of percentages may be dissimilar to 100% due to rounding. *p* < 0.05: significant difference on chi-square test

Among the patient group, those who were tested more than a year after their radiotherapy showed a higher salivary function compared to patients whose radiotherapy ended less than a year ago (*p* = 0.031). The correlations between salivary functions and texture perceptions were not significant.

## Discussion

In addition to confirming previous findings on tactile and thermal sensitivity of HNC patients [20–22], our study investigated other sub modalities of somatosensory perception. We included measurements of chemesthetic sensitivity and texture sensitivity using real food samples. We also explored the link between salivary function and sensory perception, in particular food texture sensitivity.

### Oral tactile and food texture sensitivity

The tactile sensitivity observed in the patient group is consistent with previous clinical studies employing point-pressure tests. For instance, HNC patients with hemi glossectomy were less sensitive than control but the difference is only significant when comparing the reconstructed tongue region vs. control, and not when comparing the intact tongue region vs. control [21]. Patients were less sensitive than the controls, yet the magnitude of the difference highly depends on the type of treatment and the moment at which the assessment was done (before or after treatment) [20]. Cancer patients with tumours located on the mandible and tongue/floor of mouth had a significant decrease in their tactile sensitivity following cancer treatments, but not in patients whose tumour site is on the maxillary region. The authors suggested the difference was due to the treatment site for maxillary tumours which did not involve the tongue [22]. These studies suggest that the lowered tactile sensitivity of HNC patients is attributed to the side effect of cancer treatments.

Tactile sensitivity measured using the point-pressure test is a contact-detection sensitivity which stimulates distinct parts of the slowly adapting superficial mechanoreceptors [29]. These are linked to the perception of surface properties such as roughness, particle sizes, and grittiness [30]. A reduced tactile sensitivity may translate to an altered perception of some aspects of food textures, as observed in the roughness discrimination test. A previous study demonstrated that participants with lower tactile sensitivity were shown to be less sensitive at discriminating the grittiness/roughness of chocolates [31]. The reduced sensitivity to roughness in cancer patients could also be attributed to the lack of salivation in the patient group, resulting in reduced lubrication and increased friction thereby increasing the perception of roughness [32].

Food firmness is perceived through the amount of force needed to fracture the foodstuff [33], therefore physiological factors such as jaw muscle activity and tongue function may explain the underlying difference in the firmness perception of the two groups. Radiation-induced trismus, which is the restricted mouth opening due to fibrosis of muscles, is common among HNC patients [34]. Although in this study patients who have self-reported trismus are excluded, it is not unlikely that the patients have a certain level of impairment in their jaw muscle activity [35]. Moreover, patients with cancer in the oral cavity demonstrated reduced tongue mobility and tongue force [22], altogether influencing their perception of firmness. Additionally, as the samples were semi-solids that can be masticated without chewing, the incorporation of saliva during this stage plays major importance [33, 36], thus the lack of saliva may influence the firmness perception of cancer patients.

The amount and viscosity of saliva can either dilute or intensify the perception of food thickness [37]. Thus, it was expected that cancer patients have altered sensitivity to thickness due to their reduced salivary function; however, no significant difference was observed in this study. This may be attributed to the visual bias, as the difference in visual texture was evident between the thickness samples. As sensory perception is a multidimensional process, visual appearance could also influence the judgement of textural properties [38].

### Chemesthetic and thermal sensitivity

The lower chemesthetic sensitivity may be linked to the release of inflammation-associated factors released by cancer cells which can activate and sensitise nociceptors [39]. The persistent activation may lead to chronic desensitisation of the receptors [40]. Other possible explanation may include a more acute mechanism in which the difference between patients and controls may not necessarily originate from the perceived intensity per se but from the time-intensity profile. Application or consumption of capsaicin and menthol either leads to sensitisation or desensitisation depending on the temporal delay [41]. The procedure established to evaluate the chemesthetic solutions, including the 10-s delay before evaluating the samples and the 3–4 min interstimulus interval period, was based on healthy individuals [42]. It is possible that the 10-s delay was insufficient for patients to fully perceive the sensation, or that the 3–4 min interval was too short that it caused adaptation while evaluating the proceeding samples.

Patients also demonstrated lower thermal sensitivity, consistent with previous findings [21, 22]. The authors explained that it could be attributed to the late side effects from the surgery and/or the radiotherapy which resulted in an impairment of the sensory function in the oral cavity. Medications such as NSAIDS, corticosteroids, and opioids used to treat cancer pain may also desensitise nociceptive afferents [39].

### Salivary function

The observed reduction in salivary function of cancer patients is consistent with previous findings [43–47]. Radiotherapy causes tissue damage in the radiation field. In the case of HNC, this includes severe, and sometimes permanent, damage to the salivary gland which influenced the amount and composition of saliva production [45, 46]. A reduction in parotid and submandibular glands volumes was observed 3 months after radiotherapy in the oral cavity [46], therefore reducing the salivary quantity. In addition, chemotherapeutic agents such as 5-fluorouracil and doxorubicin used by the patients also induced hyposalivation [48].

Quantity, but not quality (pH and buffering capacity) of saliva, was significantly different between the two groups. In addition to having less saliva production, cancer patients also produced thicker saliva. This may be attributed to the radiosensitivity of the different salivary glands. Parotid glands, responsible for producing most of the watery saliva, were shown to be more affected by radiation compared to submandibular glands which produce more viscous and mucin-rich saliva [43, 44, 49].

In terms of salivary quality, most of the patients were assessed more than 1 year after radiotherapy (Table 3) and had acidic saliva (pH < 6.8). Patients who were observed more than 1 year after the end of their radiotherapy showed higher salivary functions compared to those observed less than a year after the end of their radiotherapy. This is consistent with previous studies, which demonstrated a significant decrease in salivary pH after radiation but began to increase between 6 months and 2 years post-radiation, although it did not recover to the initial pH of 7.0 [45, 46]. These two longitudinal studies also showed that buffering capacity decreased upon radiation but recovered to normal at 6 months post-radiotherapy [45, 46], which also supported our findings.

In terms of food perception, saliva is an essential component influencing the perception of taste, smell, texture, temperature, and astringency [32, 50]. The lubricating property of saliva is necessary for mastication, bolus formation, and swallowing, so the lack of it may lead to eating problems [7]. The correlations between salivary function and the perception of texture were observed in a previous study [37] but in the present study, the correlations were not evident.

This study presents some limitations, for instance, the cross-sectional design does not permit to infer causality. A longitudinal study following patients across different treatments and time points would have allowed observations on the progression of their somatosensory perception. The study involved a rather heterogenous population regarding the treatment type and duration since treatment, therefore unable to discern whether the changes were caused by certain treatments or the disease itself. Further, as the test was conducted at different times of the day and periods of the year, it may influence the measurements of salivary function. Different testing locations for the two groups could potentially introduce contextual influence on perception. In addition, patients treated with radiotherapy have an enlarged periodontal ligament, which is a valuable indicator of proprioception and texture. It would therefore be interesting to study the contribution of the periodontal ligament to texture in HNC patients.

## Conclusion

The present study assessed oral somatosensory perceptions and salivary function of HNC patients, which are largely understudied relative to the taste and smell perceptions. The findings indicated that oral somatosensory alterations and salivary dysfunction are symptoms experienced by HNC patients, and the need to further explore the field. These symptoms should be carefully assessed and considered when providing nutritional support.

### Supplementary Information

Below is the link to the electronic supplementary material.

Supplementary file1 (DOCX 34 KB)

## Data Availability

The data that support the findings of this study are not openly available due to reasons of sensitivity and are available from the corresponding author upon reasonable request.

## References

[1] Sung H, Ferlay J, Siegel RL (2021). Global Cancer Statistics 2020: GLOBOCAN Estimates of Incidence and Mortality Worldwide for 36 Cancers in 185 Countries. CA Cancer J Clin.

[2] Citak E, Tulek Z, Uzel O (2019). Nutritional status in patients with head and neck cancer undergoing radiotherapy: a longitudinal study. Support Care Cancer.

[3] Wang Y, Lu Q, Zhang L (2021). Nutrition impact symptom clusters in patients with head and neck cancer receiving concurrent chemoradiotherapy. J Pain Symptom Manage.

[4] Farhangfar A, Makarewicz M, Ghosh S (2014). Nutrition impact symptoms in a population cohort of head and neck cancer patients: multivariate regression analysis of symptoms on oral intake, weight loss and survival. Oral Oncol.

[5] Kathrine A, Christine L, Mathilde T (2021). Taste alterations and oral discomfort in patients receiving chemotherapy. Support Care Cancer.

[6] Langius JAE, Doornaert P, Spreeuwenberg MD (2010). Radiotherapy on the neck nodes predicts severe weight loss in patients with early stage laryngeal cancer. Radiother Oncol.

[7] Galaniha LT, Nolden AA (2022). The role of saliva in taste dysfunction among cancer patients: mechanisms and potential treatment. Oral Oncol.

[8] Hutton JL, Baracos VE, Wismer WV (2007). Chemosensory dysfunction is a primary factor in the evolution of declining nutritional status and quality of life in patients with advanced cancer. J Pain Symptom Manage.

[9] Brisbois TD, De Kock IH, Watanabe SM (2011). Characterization of chemosensory alterations in advanced cancer reveals specific chemosensory phenotypes impacting dietary intake and quality of life. J Pain Symptom Manage.

[10] García-Peris P, Parón L, Velasco C (2007). Long-term prevalence of oropharyngeal dysphagia in head and neck cancer patients: impact on quality of life. Clin Nutr.

[11] Dalton J, Rothpletz-Puglia P, Epstein JB (2022). Transitioning the eating experience in survivors of head and neck cancer. Support Care Cancer.

[12] Ganzer H, Rothpletz-Puglia P, Byham-Gray L (2015). The eating experience in long-term survivors of head and neck cancer: a mixed-methods study. Support Care Cancer.

[13] Small DM (2012). Flavor is in the brain. Physiol Behav.

[14] Hollins M (2010). Somesthetic senses. Annu Rev Psychol.

[15] Chen J (2014). Food oral processing: some important underpinning principles of eating and sensory perception. Food Struct.

[16] Pedersen AM, Bardow A, Jensen SB, Nauntofte B (2002). Saliva and gastrointestinal functions of taste, mastication, swallowing and digestion. Oral Dis.

[17] Haahr AM, Bardow A, Thomsen CE (2004). Release of peppermint flavour compounds from chewing gum: effect of oral functions. Physiol Behav.

[18] Gunn L, Gilbert J, Nenclares P (2021). Taste dysfunction following radiotherapy to the head and neck: a systematic review. Radiother Oncol.

[19] Álvarez-Camacho M, Gonella S, Campbell S (2017). A systematic review of smell alterations after radiotherapy for head and neck cancer. Cancer Treat Rev.

[20] Bodin I, Jäghagen EL, Isberg A (2004). Intraoral sensation before and after radiotherapy and surgery for oral and pharyngeal cancer. Head Neck.

[21] Loewen IJ, Boliek CA, Harris J (2010). Oral sensation and function: a comparison of patients with innervated radial forearm free flap reconstruction to healthy matched controls. Head Neck.

[22] de Groot RJ, Merkx MAW, Hamann MNS (2020). Tongue function and its influence on masticatory performance in patients treated for oral cancer: a five-year prospective study. Support Care Cancer.

[23] Burges Watson DL, Lewis S, Bryant V (2018). Altered eating: a definition and framework for assessment and intervention. BMC Nutr.

[24] Crowder SL, Douglas KG, YaninaPepino M (2018). Nutrition impact symptoms and associated outcomes in post-chemoradiotherapy head and neck cancer survivors: a systematic review. J Cancer Surviv.

[25] Cattaneo C, Liu J, Bech AC (2020). Cross-cultural differences in lingual tactile acuity, taste sensitivity phenotypical markers, and preferred oral processing behaviors. Food Qual Prefer.

[26] Nolden AA, Hayes JE (2017). Perceptual and affective responses to sampled capsaicin differ by reported intake. Food Qual Prefer.

[27] Elfring TT, Boliek CA, Seikaly H (2012). Sensory outcomes of the anterior tongue after lingual nerve repair in oropharyngeal cancer. J Oral Rehabil.

[28] Bearelly S, Wang SJ, Cheung SW (2017). Oral sensory dysfunction following radiotherapy. Laryngoscope.

[29] Abraira VE, Ginty DD (2013). The sensory neurons of touch. Neuron.

[30] Engelen L, Van Der Bilt A (2008). Oral physiology and texture perception of semisolids. J Texture Stud.

[31] Breen SP, Etter NM, Ziegler GR, Hayes JE (2019). Oral somatosensatory acuity is related to particle size perception in chocolate. Sci Rep.

[32] De Wijk RA, Prinz JF (2006). Mechanisms underlying the role of friction in oral texture. J Texture Stud.

[33] Devezeaux de Lavergne M, van de Velde F, van Boekel MAJS, Stieger M (2015). Dynamic texture perception and oral processing of semi-solid food gels: Part 2: Impact of breakdown behaviour on bolus properties and dynamic texture perception. Food Hydrocoll.

[34] Abboud WA, Hassin-Baer S, Alon EE (2020). Restricted mouth opening in head and neck cancer: etiology, prevention, and treatment. JCO Oncol Pract.

[35] Martins CA, Goldenberg DC, Narikawa R, Kowalski LP (2020). Trismus and oral health conditions during diagnosis of malignant oral neoplasms. Braz J Otorhinolaryngol.

[36] Engelen L, de Wijk RA (2012) Oral processing and texture perception. In: Chen J, Engelen L (eds) Food oral processing. Wiley‐Blackwell, Oxford, pp 157–176

[37] Engelen L, van den Keybus PAM, de Wijk RA (2007). The effect of saliva composition on texture perception of semi-solids. Arch Oral Biol.

[38] Spence C (2017) Multisensory flavour perception. In: Guichard E, Salles C, Morzel M, Le Bon A-M (eds) Flavour: from food to perception. John Wiley & Sons, Incorporated, United Kingdom, pp 373–394

[39] Mantyh PW, Clohisy DR, Koltzenburg M, Hunt SP (2002). Molecular mechanisms of cancer pain. Nat Rev Cancer.

[40] Alsalem M, Millns P, Altarifi A (2016). Anti-nociceptive and desensitizing effects of olvanil on capsaicin-induced thermal hyperalgesia in the rat. BMC Pharmacol Toxicol.

[41] Cliff MA, Green BG (1996). Sensitization and desensitization to capsaicin and menthol in the oral cavity: interactions and individual differences. Physiol Behav.

[42] Green BG (1991). Temporal characteristics of capsaicin sensitization and desensitization on the tongue. Physiol Behav.

[43] Li Y, Taylor JMG, Ten Haken RK, Eisbruch A (2007). The impact of dose on parotid salivary recovery in head and neck cancer patients treated with radiation therapy. Int J Radiat Oncol Biol Phys.

[44] Murdoch-Kinch CA, Kim HM, Vineberg KA (2008). Dose-effect relationships for the submandibular salivary glands and implications for their sparing by intensity modulated radiotherapy. Int J Radiat Oncol Biol Phys.

[45] Lin CY, Ju SS, Chia JS (2015). Effects of radiotherapy on salivary gland function in patients with head and neck cancers. J Dent Sci.

[46] Sim CPC, Soong YL, Pang EPP (2018). Xerostomia, salivary characteristics and gland volumes following intensity-modulated radiotherapy for nasopharyngeal carcinoma: a two-year follow up. Aust Dent J.

[47] Barbosa da Silva JL, Doty RL, Miyazaki JVMK (2019). Gustatory disturbances occur in patients with head and neck cancer who undergo radiotherapy not directed to the oral cavity. Oral Oncol.

[48] Jensen SB, Pedersen AM, Reibel J, Nauntofte B (2003). Xerostomia and hypofunction of the salivary glands in cancer therapy. Support Care Cancer.

[49] Deasy JO, Moiseenko V, Marks L (2010). Radiotherapy dose-volume effects on salivary gland function. Int J Radiat Oncol Biol Phys.

[50] Lester S, Hurst K, Cornacchia L (2021). The relation between stimulated salivary flow and the temporal consumption experience of a liquid oral nutritional supplement. Appetite.

